# Regulation of the Immune System Development by Glucocorticoids and Sex Hormones

**DOI:** 10.3389/fimmu.2021.672853

**Published:** 2021-06-23

**Authors:** Linda Quatrini, Biancamaria Ricci, Cecilia Ciancaglini, Nicola Tumino, Lorenzo Moretta

**Affiliations:** Department of Immunology, IRCCS Bambino Gesù Children’s Hospital, Rome, Italy

**Keywords:** glucocorticoids, sex hormones, hematopoietic stem cell transplantation, hematopoietic stem and progenitor cell, immune system development

## Abstract

Through the release of hormones, the neuro-endocrine system regulates the immune system function promoting adaptation of the organism to the external environment and to intrinsic physiological changes. Glucocorticoids (GCs) and sex hormones not only regulate immune responses, but also control the hematopoietic stem cell (HSC) differentiation and subsequent maturation of immune cell subsets. During the development of an organism, this regulation has long-term consequences. Indeed, the effects of GC exposure during the perinatal period become evident in the adulthood. Analogously, in the context of HSC transplantation (HSCT), the immune system development starts *de novo* from the donor HSCs. In this review, we summarize the effects of GCs and sex hormones on the regulation of HSC, as well as of adaptive and innate immune cells. Moreover, we discuss the short and long-term implications on hematopoiesis of sex steroid ablation and synthetic GC administration upon HSCT.

## Introduction

**Figure 1:**
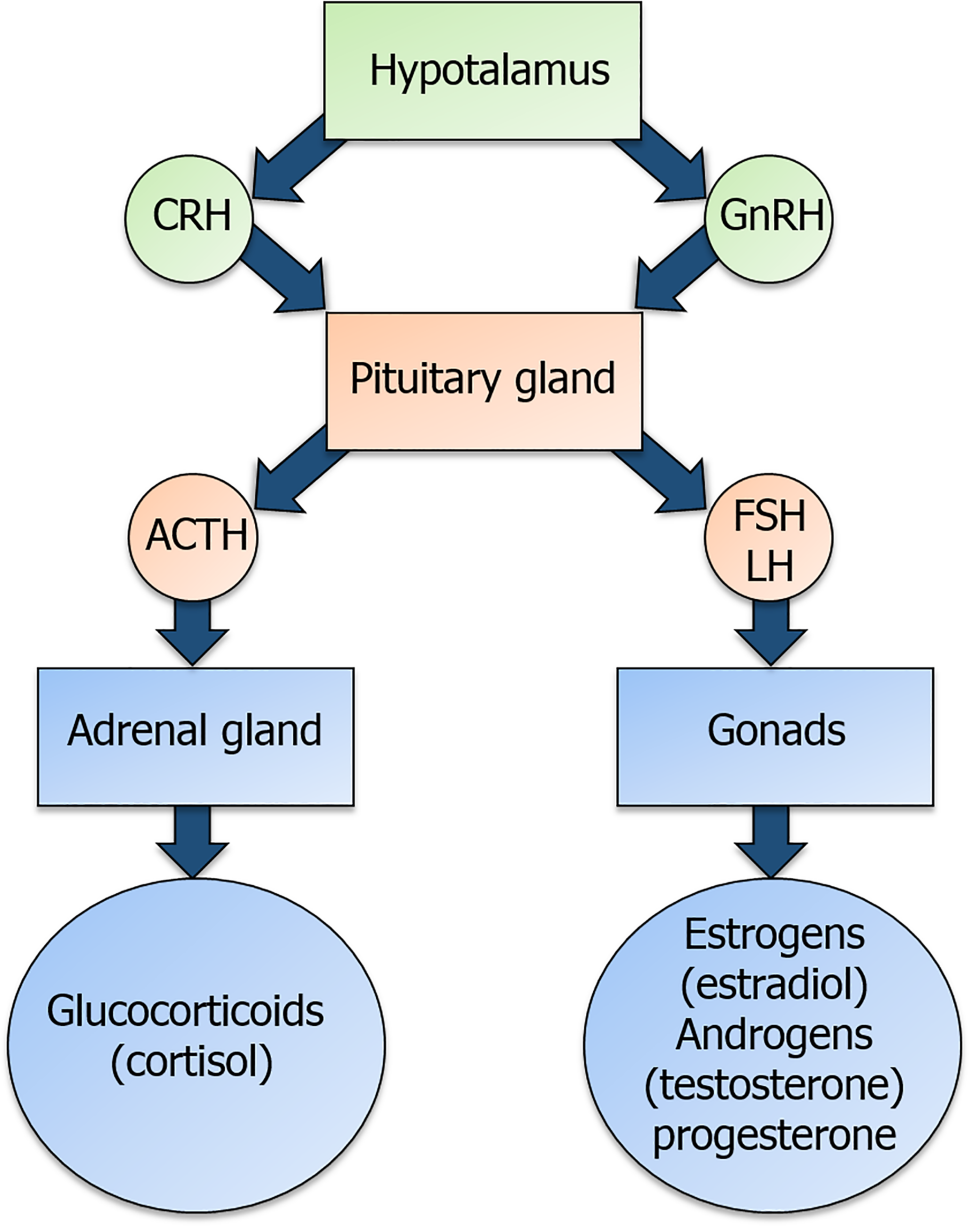
Overview of the hypothalamic-pituitary-adrenal (HPA) axis and of the hypothalamic-pituitary-gonadal (HPG) axis. Corticotropin-releasing hormone (CRH) and gonadotropin-releasing hormone (GnRH) are released by the hypothalamus and act on their receptors in the pituitary gland. In detail, CRH binding to its receptor triggers the production of the adrenocorticotropic-hormone (ACTH), while GnRH binding to its receptor triggers the production of the gonadotropins follicle-stimulating hormone (FSH) and luteinizing hormone (LH). ACTH and gonadotropins are released in the bloodstream and act on the adrenal gland and the gonads, respectively, to induce the secretion of glucocorticoids and sex hormones.

Glucocorticoids (GCs) and the sex hormones estrogens, progesterone and androgens are produced under the control of the hypothalamic-pituitary-adrenal (HPA) and hypothalamic-pituitary-gonadal (HPG) axis, respectively, through a common steroidogenic pathway from cholesterol (1) (**Figure 1**). GC synthesis occurs in the cortical part of the adrenal gland for both males and females. Androgens are produced in male gonads and, in smaller amounts, in the ovary and adrenal cortex in females. Estrogens are mainly produced from androgens precursors in ovarian granulosa cells and placenta in females, testis in males and non-glandular tissue (fat and bone) in both sexes (2). Progesterone is produced by ovarian granulosa cells, adrenal glands, corpus luteum during the menstrual cycle and placenta (**Figure 1**) in females and by adrenal glands in both males and females (3).

GCs are released into circulation in response to the circadian cycle, are essential regulators of body functions in homeostasis and allow adaptation to the environmental changes. Moreover, due to their potent immune-suppressive and anti-inflammatory effects, synthetic GCs are widely used in clinics to treat acute and chronic inflammation (4). On the other hand, besides regulating reproductive and metabolic body functions throughout the lifespan, sex hormones are also able to regulate immune response and inflammation. Indeed, a key example is given by the predominance of autoimmune diseases in females (5) and by sex dimorphism in antitumor immunity and response to infections (6, 7). In line with this, it was shown that estrogen-dependent protection of women from hepatitis C virus and liver cancer incidence are lost after menopause (8, 9). Age- and sex-related changes in immune response have therefore clinical consequences and should be taken into account because they may affect the efficacy of vaccination and cancer immunotherapy (10).

Because of their lipophilic nature, steroid hormones can cross cell membranes and bind to nuclear receptors belonging to the same superfamily of the ligand-regulated transcription factors (TFs) (11). In particular, they are the GC receptor (GR) and the sex hormone receptors estrogen receptor (ER) ERα and ERβ, progesterone receptor (PR), and androgen receptor (AR). These receptors are characterized by polymorphisms and multiple isoforms, responsible for variations in individual response at both molecular and clinical level. Steroid hormone receptors possess a N-terminal transactivation domain, a conserved central DNA binding domain and a C-terminal ligand-binding domain. In the cytoplasm, they are bound to chaperone proteins (e.g., HSP90). The binding of the ligand frees the receptor from the chaperone, allowing homodimerization, exposure of the nuclear localization sequence, and entry into the nucleus. Once in the nucleus, the ligand–receptor complex can directly bind to DNA sequences termed hormone response elements, and can associate with transcriptional coactivators to facilitate the regulation of target genes. It has been shown that steroid hormones can also mediate rapid “non-genomic” effects by acting directly on cytoplasmatic signal transduction pathways (12, 13). In addition, steroid hormones can alter gene expression through their effects on epigenetic modifications (14–16). For example, GR represses IL-5 transcription by recruiting and interacting with the Histone Deacetylase 1 (HDAC1) (17), and can inhibit p65-mediated transcription by recruiting HDAC2 (18).

Thus, the steroid hormone receptor-driven transcriptional regulation in a given cellular context may be mediated by at least two non-mutually exclusive mechanisms: chromatin remodeling and partnering with a specific repertoire of TFs (19).

Immune cells express receptors for GCs and sex hormones and are therefore regulated by these steroids. However, the response depends on many factors, including the developmental stage, the activation state and the inflammatory signals the cell receives at the same time. In this review, we will summarize the effects of GCs and sex hormones on the development of the immune system and how this regulation affects the immune response in the long term (**Table 1**).

**Table 1:** Summary of the main effects of GCs and sex hormones on HSC, adaptive and innate immune cells.

	Hormone or hormone receptor	Target cell	Effect	Reference
Hematopoietic stem cells	GR	erythroid progenitor	increased self-renewal	(20)
	GR	HSC	upregulation of CXCR4 expression and homing to the BM	(21)
	LH	HSC	regulation of cell expansion	(22)
	ERα	HSC	increased self-renewal	(23)
	ERα	HSC	increased telomerase activity	(24)
Adaptive immune cells	GC	thymocytes	apoptosis	(25)
	GR	CD8+ T cell	generation of memory precursor cells	(26)
	DT	thymocytes	TNFα-mediated apoptosis	(27)
	ERα	thymocytes	thymic atrophy	(28)
	AR	cTEC	inhibition of Dll4 expression	(29)
	AR	mTEC	upregulation of Aire expression	(30)
	ERα	mTEC	downregulation of Aire expression	(31)
	ERα	lymphoid precursors	decrease of B lymphopoiesis	(32)
	E2	lymphoid precursors	cell depletion in the BM	(33)
	AR	BM stromal cell	defect in B lymphopoiesis	(34)
Innate immune cells	GC	HSC	induction of NK cell differentiation from myeloid precursors	(35)
	GC	monocytes	block of differentiation towards DCs	(36)
	GC	DCs	impairment of DCs terminal maturation	(37)
	ERα	myeloid precursors	inhibition of Flt3-induced conventional and plasmacytoid DC development	(38)
	ERα	myeloid precursors	increased GM-CSF-induced DC differentiation	(39)

GR, glucocorticoid receptor; HSC, hematopoietic stem cell; BM, bone marrow; LH, luteinizing hormone; ER, estrogen receptor; GC, glucocorticoid; DT, depo-testosterone; TNF, tumor necrosis factor; AR, androgen receptor; cTEC, cortical thymic epithelial cells; mTEC, medullary thymic epithelial cells; Dll4, Delta-like 4; E2, estradiol; NK, natural killer; DC, dendritic cell; Flt3, Fms related tyrosine kinase 3; GM-CSF, Granulocyte macrophage-colony stimulating factor.

## GCs and Sex Hormones Regulation of HSC Development

Hematopoietic stem cells (HSCs) are multipotent self-renewing units mediating the generation of all blood constituents. An important site for the development of HSC in the embryo is the dorsal aorta, which contains a transient population of blood-producing endothelial cells called hemogenic endothelium (40). Studies on the role of GCs on the early development of immune system in the embryo were done in a zebrafish experimental model (41). It was shown that, upon stress, the central nervous system releases serotonin that, in turn, activates the HPA axis. GC release controls HSC specification promoting hematopoietic stem and progenitor cell (HSPC) formation in the dorsal aorta of embryos (41). These findings suggest that any stress that may be experienced by the embryo during development, such as hypoxia, temperature changes, metabolic or oxidative stress, would promote blood cell formation through activation of the HPA axis.

After birth, steroid hormones control the formation of immune cells by acting on HSCs in the bone marrow (BM). Studies have been performed in mouse models and on *ex vivo* cultures of human CD34^+^ HSCs to investigate this matter, especially with the purpose of developing therapeutic strategies to treat hematological diseases (42). For instance, it was suggested that GC treatment may be efficient to treat erythropoietin-resistant anemias because GR signaling regulates erythropoiesis. In particular, it was shown that GR synergizes with peroxisome proliferator-activated receptor α (PPAR-α) to induce self-renewal of erythroid progenitor cells *in vivo* and increase erythroid cell expansion *ex vivo* from human CD34^+^ HSCs (20).

HSC transplantation (HSCT) represents the common therapeutic approach to treat malignant and non-malignant hematological disorders. G-CSF-mediated mobilization of HSC has emerged as the most suitable means to obtain HSCs for transplantation, complemented by plerixafor (a selective CXCR4 antagonist) treatment in “poor-mobilizers” (43, 44). In mice, a neuroendocrine pathway that promotes HSC migration from the BM to the peripheral blood has been identified. It was shown that cholinergic signals through the muscarinic receptor type-1 (Chrm1) in the brain trigger HPA axis activation and GC production. GR signaling in HSC induces an up-regulation of actin-organizing molecules, thus promoting cell migration (45). HSC homing to the BM upon intravenous injection is mediated by coordinated actions of adhesion receptors and the chemokine CXCL12 expressed in the BM microenvironment (46). In a screen of small molecule compounds, GCs were identified as activators of CXCR4 expression in human CD34^+^ HSC isolated from cord blood (CB). It was shown that short term GC pretreatment is able to induce GR binding to a GC-response element in the CXCR4 promoter and SRC-1-p300 complex recruitment to induce H4K5 and H4K16 histone acetylation, favoring gene transcription. GC-induced CXCR4 up-regulation mediated HSC chemotaxis in response to CXCL12 and homing in the BM. Notably, although GC pretreatment was short, long-term engraftment was observed upon HSC transplantation into primary- and secondary-recipient NSG mice (21).

The regulation of HSC homeostasis by the sex hormones takes place after birth and affects their number and self-renewal capacity at different developmental stages. Luteinizing hormone (LH) is secreted by the pituitary gland at the onset of puberty, promoting the maturation of the reproductive system in both males and females. In post-natal BM, HSCs are a direct target of LH, whose signaling acts like a brake of cell overexpansion, ensuring a proper HSC count for normal hematopoiesis in adulthood (22). HSCs from mice treated with the estrogen estradiol (E2) have increased regenerative capacity after transplantation or irradiation, thus explaining why female immunodeficient recipient mice support reconstitution of the blood system by transplanted human HSCs more efficiently than the male counterpart (47). It was suggested that E2-ERα signaling in HSCs induces the expression of Ern1, which encodes Ire1a, thus activating the Ire1a-Xbp1 pathway of the unfolded protein response and promoting HSC resistance against proteotoxic stress (48). It was shown that Tamoxifen (a selective ER modulator) by signaling through ERα has a minor impact on primitive normal HSCs, but induces apoptosis in malignant HSCs enhancing chemotherapy response in a mouse model of acute myeloid leukemia (49). During pregnancy, ERα signaling in HSCs increases cell division, frequency, and erythropoiesis in the spleen of female mice (23). In particular, E2 promotes increased HSC self-renewal in the bone marrow, while another endogenous ligand for ERα (27-Hydroxycholesterol-27HC) promotes HSC mobilization to the spleen (50). Thus, these two ERα agonists collaborate to favor extramedullary hematopoiesis, required during pregnancy to maintain red blood cell counts despite a rapidly increasing blood volume.

Impaired DNA repair, altered DNA methylation patterns, aberrant metabolism and skewed upregulation of myeloid- (at the expense of lymphoid-) associated genes contribute to altering HSC functions with age (51). Both intrinsic functional changes in the earliest HSCs and extrinsic alterations of the HSC niche contribute to this degeneration (52). Evidence suggests that sex steroids play at least some roles in age-related decline of the immune functions (53) which, indeed, becomes more evident from the onset of puberty, when the levels of these hormones increase (54, 55). A comparison of healthy men and women’s PBMCs revealed a shared epigenomic signature of aging, including declining naïve T cell and increasing monocyte and cytotoxic cell functions. Age-related epigenomic changes first spike around late-thirties with similar timing and magnitude between sexes, whereas the second spike occurs earlier and stronger in men (56). Consistent with a role for sex steroids in the decline of the immune functions, sex steroid ablation (SSA) today represents an attractive therapeutic approach to restore immune competence in immunodeficient individuals (57). It has been shown that SSA has the potential to accelerate the immune recovery in many clinical conditions such as upon autologous and allogeneic HSCT, or after damaging cytoablative treatments like chemotherapy. SSA consists in therapeutically targeting the upstream signaling events such as LH releasing hormone (LHRH) or directly blocking sex steroid receptors (58, 59). This has been achieved clinically for over 35 years because, in addition to its well established role in improving thymopoiesis (see following section), several studies have found that SSA contrasts the age-related decline of early lymphoid progenitors in the BM. SSA does not enhance HSC homing to the BM, but increases their self-renewal and lymphoid differentiation capacities (60). These effects are due to intrinsic changes in HSCs, as well as to an extrinsic effect on the stromal microenvironment. In particular, SSA induces the upregulation of genes implicated in the protection from aging such as Foxo1 in the hematopoietic niche thus increasing its ability to support hematopoiesis (60). Interestingly, while SSA through direct and indirect mechanisms contrasts aging of early lymphoid progenitors, androgens have been shown to increase telomerase activity in primary HSCs (24). For its therapeutic effect on erythropoiesis, the androgen derivative Danazol is now used in the treatment of acquired severe aplastic anemia (61).

## GCs and Sex Hormones Control the Development of the Adaptive Immune System

The thymus is a critical site for generating a diverse T cell repertoire while maintaining self-tolerance, and both GCs and sex hormones play a critical role in shaping thymic functions. In particular, GCs are crucial in the selection of the appropriate T cell receptor (TCR) self-affinities repertoire in the thymus (62). By dampening the strength of TCR signals, GCs prevent negative selection of T cells that have high affinity for self MHC (63). On the other hand, double positive thymocytes are the most sensitive to GC-induced apoptosis, and indeed stress response inducing GC production (including psychological stress, fasting, injury and infection) causes an acute reduction in thymus size (25, 64, 65). GCs also regulate the subsequent thymus-independent steps of T cell development. A characterization of the epigenetic landscapes of naïve, terminal-effector, memory precursor and memory CD8^+^ T cells revealed that the expression and binding of specific TFs contribute to the establishment of subset-specific enhancers during differentiation, which control the *in vivo* response to bacterial infections (26). One of these key TF is Nr3c1, encoding for GR, constitutively expressed during CD8^+^ T cell differentiation and involved in the generation of memory precursor cells (26). GR has an important role in T cell differentiation not only in physiological, but also in pathological conditions. Indeed, in tumor infiltrating lymphocytes, an increasing gradient of GR expression and signaling from naïve to dysfunctional CD8^+^ T cells was found, suggesting that endogenous GCs also promote T cell dysfunction and tumor growth (66).

Testosterone can directly induce apoptosis of double positive thymocytes through the upregulation of TNF-α, while estrogens can induce thymic atrophy by eliminating early thymic progenitors and inhibiting the proliferation of β-selected thymocytes (27, 28). They have also an indirect effect on thymopoiesis since the expression of sex steroid receptor is significantly higher on thymic epithelia cells than on thymocytes. Indeed, sex steroids inhibit in cortical thymic epithelial cells (cTECs) the expression of Delta-like 4 (Dll4), a Notch ligand crucial for the commitment and differentiation of T cell progenitors in a dose-dependent manner (29). It was demonstrated that androgen-response elements are present in the promoter of the Dll4 gene and androgen/AR complexes were localized to the Dll4 promoter by chromatin immunoprecipitation (29). Sex hormones also control the expression of Aire (Autoimmune Regulator), the transcription factor responsible for the expression of thousands of tissue-restricted proteins in cells deputed to the presentation of self-antigens to the maturing T lymphocytes. By upregulating Aire expression in medullary TEC (mTEC) by directly binding its promoter, androgens enforce self-tolerance and lead to a more efficient negative selection of self-reactive T cells (30). At the same time, estrogens can decrease Aire expression by epigenetically regulating its methylation (31). Indeed, Aire expression in mice and human thymus is higher in males compared to females, suggesting that increased levels of androgens and decreased levels of estrogens protect males from autoimmunity by promoting Aire expression. All of these molecular mechanisms identified for sex hormones explain why SSA increases thymic cellularity, restores thymic architecture and organization, and enhances thymopoiesis in young and adult animals (58).

Age-related immune system decline is not only restricted to thymic atrophy, but also to an intrinsic defect in the HSC ability to commit to the lymphoid lineage. Therefore, with age there is a significant decline in B lymphopoiesis and humoral immunity, accompanied by a reduced peripheral immune repertoire, leading to increased opportunistic infections and limited recovery following cytoablation after chemo or radiotherapy (67). Estrogens reduce responsiveness to IL-7 causing decreased B lymphopoiesis (32) and deplete lymphoid committed precursors in the BM (33). Androgens affect B lymphopoiesis indirectly, by acting on BM stromal cells (34). Following surgical SSA in mice displaying age- or chemotherapy-induced immunodepletion, a rapid and sustained increase in developing B cells and their upstream lymphoid progenitors was observed. SSA not only increased B cell number, but also enhanced humoral response to challenge by hepatitis B vaccine (68).

## GCs and Sex Hormones Regulation of Innate Immune Cells

Innate lymphoid cells (ILCs) differentiate from HSC in the BM, and common ILC precursors continue their development in secondary lymphoid tissues (69). GC treatment of human CB CD34^+^ HSCs accelerated Natural Killer (NK) cell differentiation, promoted a switch of myeloid precursors towards immature NK cells and induced NK cell cytolytic activity (35). Although a role for the neuroendocrine system in the regulation of helper ILC functions has been shown (70), its contribution to their development has not been investigated so far.

In mice, GC exposure favors myelopoiesis in the bone marrow, suggesting that preservation of granulocytes and their progenitors may be a mechanism to ensure rapid protection of the organism upon stress (71). Neutrophil development and function are modulated also by sex hormones. In particular, healthy young adult women display an activated/mature neutrophil profile characterized by enhanced type I IFN pathway activity, enhanced proinflammatory responses, and distinct immunometabolism compared to young men (72).

Blood monocyte precursors originate in the BM, enter the circulation and are present in the blood until they migrate into tissues where they can differentiate into macrophages and dendritic cells (DC) (73). GCs are long known to act on monocytes influencing their short time mediator release, but there is growing evidence that they are also involved in differentiation processes skewing towards an anti-inflammatory phenotype (74). GCs block the generation of immature DCs from monocytes (36) and impair terminal maturation of already differentiated DCs (37). Similarly, ER signaling inhibits Flt3-induced conventional and plasmacytoid DC development from myeloid precursors (38). On the contrary, it was shown that GM-CSF-induced DC differentiation as Langerhans cells from myeloid progenitors was promoted by adding E2 *in vitro* and inhibited by ER antagonists and ERα-deficiency (39). Indeed, ERα signaling during this process targets IRF4, a TF critical for Langerhans cell development (75).

Interestingly, mast cell cytokine production seems to be sex dependent. Indeed, in females they mainly produce proinflammatory cytokines, such as TNF and IL-1β, while their activation in males results in a predominant production of IL-33 (76). Although both male and female derived mast cells express AR, testosterone induces IL-33 production only in male-derived cells *in vitro* (77). These data suggest that, in addition to a direct effect, testosterone may have other effects during development that poise mast cells for IL-33 expression potential, perhaps at an epigenetic level. Therefore, the ability to express a particular array of cytokines may be programmed by prolonged exposure to sex hormones starting early in life and is not solely due to acute sex hormone-receptor interactions.

## Conclusions and Future Perspectives: A Role for GCs and Sex Hormones in the Developmental Programming of Immune System?

Early life exposure to environmental cues, particularly during the perinatal period, can have a life-long impact on the organism development and physiology. The biological rationale for this phenomenon is to promote physiologic adaptation to the anticipated environment based on early life experience. A role in this “developmental programming” for early life stress or prenatal treatment with synthetic GCs during sensitive windows of development has been established (78). Excessive or premature exposure to GCs has been associated to long term effects on tissues and organs (hypertension, hyperglycemia, anxiety), as well as to higher risks for atopic diseases, asthma, autoimmune type I diabetes, infectious diseases and decreased adaptive and anti-tumor immune response (79–83). GC-induced programming of the immune system is mediated by effects on HSCs or other persistent progenitors, which endure through the individual’s lifespan (84, 85), rather than upon the short-lived fully differentiated immune cell populations. The long-term consequences of perinatal GC exposure were recently evaluated in mice in a study by putting the synthetic GC dexamethasone in the drinking water from mid-pregnancy to early post-natal period, when hematological and lymphoid organogenesis takes place (86). As a result, the authors observed a diminished CD8^+^ T cell response in adulthood and impaired control of tumor growth and bacterial infections. Perinatal GC exposure led to HPA axis reprogramming with alterations in its threshold, decreased systemic levels of GCs and persistent changes in the chromatin state of naïve T cells (86). The gene expression program was “imprinted”, as the changes were maintained also upon cell adoptive transfer to new environments and, importantly, the stem cell compartment contributed to compromised T cell responses, as the defect could be observed also in irradiated mice transplanted with the BM of GC-treated mice (86).

These findings suggest that GCs, as well as the other hormones whose receptors function as TFs, may be able to induce an “imprinting” in HSC gene expression program, most likely at the epigenetic level, thus stably affecting HSC developmental trajectories. This kind of regulation that occurs during organism development may be recapitulated upon HSCT when the immune system development from donor-derived HSCs starts *de novo*. Since SSA and GCs are widely used to improve HSCT outcome and prevent complications such as graft *vs* host disease, respectively, it would be interesting to gain further insights into the short and long term consequences of these treatments on hematopoiesis. A better understanding of HSC regulation by GCs and sex hormones is extremely important to predict their effects on the immune system development and design therapeutic approaches to improve the long-term reconstitution in HSCT recipients.

## Author Contributions

All authors contributed to the article and approved the submitted version.

## Funding

This work was supported by grants awarded by Associazione Italiana per la Ricerca sul Cancro (AIRC)-Special Program Metastatic disease: the key unmet need in oncology 5X1000 2018 Id. 21147 (LM), AIRC IG 2017 Id. 19920 (LM), and RC-2020 OPBG (LM). LQ has received funding from AIRC and from the European Union’s Horizon 2020 research and innovation programme under the Marie Skłodowska-Curie grant agreement no 800924.

## Conflict of Interest

The authors declare that the research was conducted in the absence of any commercial or financial relationships that could be construed as a potential conflict of interest.
